# Mature vessel occlusion after anti-VEGF treatment in a retinal arteriovenous malformation

**DOI:** 10.1186/1471-2415-13-60

**Published:** 2013-10-21

**Authors:** Lan-Hsin Chuang, Nan-Kai Wang, Yen-Po Chen, Wei-Chi Wu, Chi-Chun Lai

**Affiliations:** 1 Department of Ophthalmology, Chang-Gung Memorial Hospital, Keelung, Taiwan; 2 Department of Ophthalmology, Chang-Gung Memorial Hospital, Linkou, Taiwan; 3 Chang Gung University College of Medicine, Taoyuan, Taiwan

## Abstract

**Background:**

To report engorged vessel occlusion after repeated intravitreal injections of bevacizumab to treat the macular oedema in a case of arteriovenous malformation.

**Case presentation:**

A 37-year-old woman presented with a sudden, painless loss of vision in her left eye. Her visual acuity was 20/200 in the left eye, and 20/20 in the right eye. Ophthalmoscopic examination revealed an abnormal tangle of vessels and enlarged draining veins. A fluorescence angiogram revealed fluorescence leakage at a turn near the fovea. Horizontally oriented optical coherence tomography revealed an increased macular thickness and an accumulation of intraretinal fluid, indicating macular oedema. After three intravitreal injections of 1.25 mg bevacizumab, her vision improved to 20/20. Ophthalmoscopic examination revealed a decreased calibre of the previously engorged draining veins and ghost vessels. Repeated horizontally oriented optical coherence tomography revealed a decreased macular thickness and the absence of an intraretinal cyst. At the 2-year follow-up visit, the vision of the patient was stable.

**Conclusion:**

This finding implies that certain middle-size vessels can become occluded during anti- vascular endothelium growth factor (anti-VEGF) therapy, which could induce fatal complications if it occurred in the heart or brain. Clinicians should be cautious of the potential thrombotic effects on systemic blood vessels when administering anti-VEGF treatment.

## Background

Anti-vascular endothelium growth factor (anti-VEGF) agents have antiangiogenic and oedema-diminishing actions and are used widely to diminish new vessels and reduce the oedema of ocular diseases [1-4]. Anti-VEGF is believed to diminish new vessels but not mature vessels and is therefore considered to be a relatively safe therapy [1-4]. Herein, we report the first case of a grade 1 retinal arteriovenous (AV) malformation [5] in which the patient regained vision after repeated intravitreal injections of bevacizumab. Unexpectedly, we observed occlusion of the engorged vessels after repeated intravitreal injections of bevacizumab to treat the macular oedema.

## Case presentation

**Figure 1:**
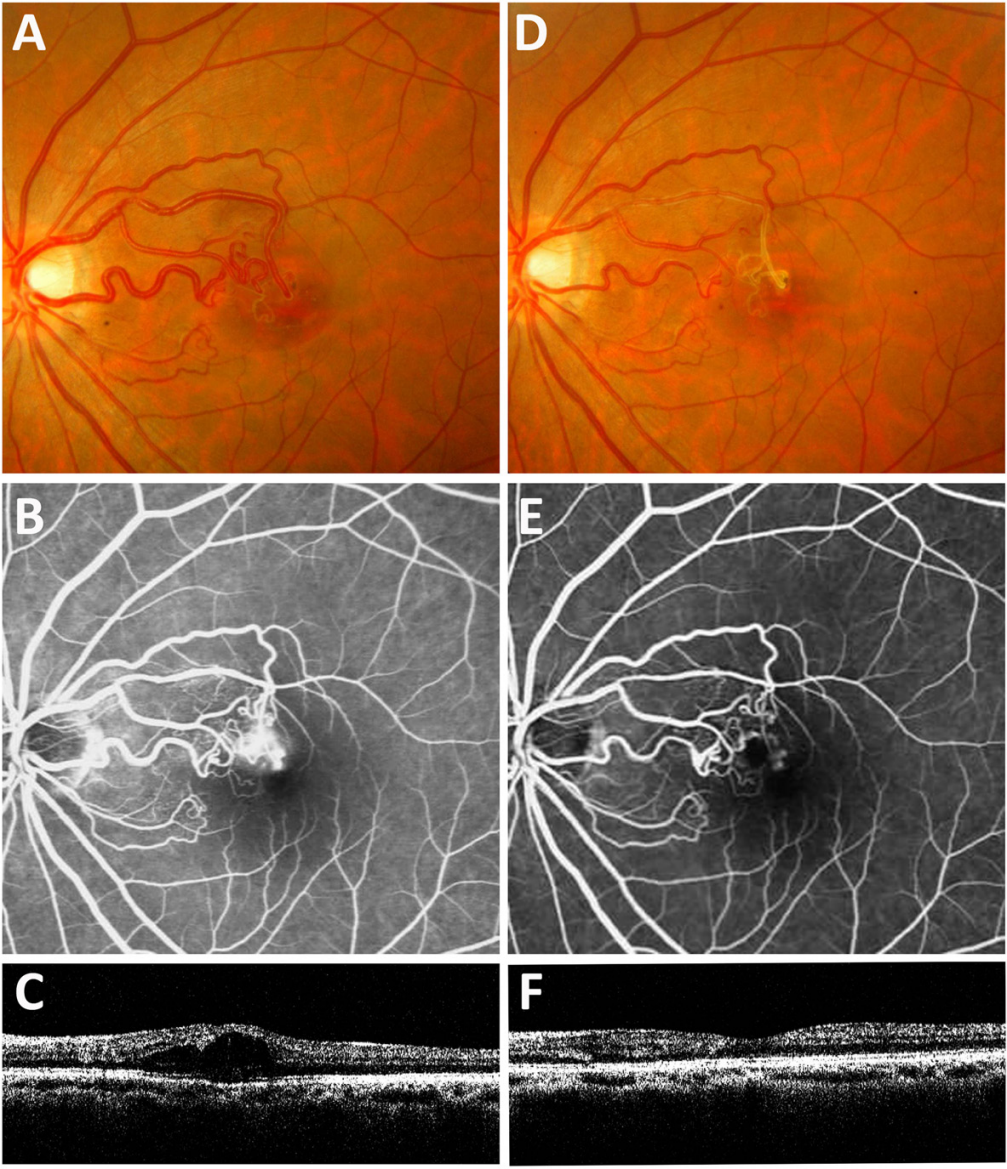
**Fundus photo, fluorescence angiography and optical coherent tomography of patient. (A)**. Ophthalmoscopic examination revealed an abnormal tangle of vessels and enlarged draining veins. **(B)**. Fluorescence angiography revealed fluorescence leakage at a turn near the fovea. **(C)**. Horizontally oriented optical coherence tomography revealed an increased macular thickness and an accumulation of intraretinal fluid, indicating macular oedema. **(D)**. Six months after the third intravitreal injection of bevacizumab, ophthalmoscopic examination revealed a decreased calibre of the previously engorged draining veins and ghost vessels. **(E)**. Fluorescence angiography revealed the absence of leakage at the fovea and a decreased calibre in the occluded arteries. **(F)**. Horizontally oriented optical coherence tomography revealed a decreased macular thickness and the absence of an intraretinal cyst.

A 37-year-old woman presented with a sudden, painless loss of vision in her left eye. Her visual acuity was 20/200 in the left eye and 20/20 in the right eye. Ophthalmoscopic examination revealed an abnormal tangle of vessels and enlarged draining veins (Figure 1A); these signs were compatible with the diagnosis of a grade 1 retinal AV malformation. Fluorescence angiography revealed fluorescence leakage at a turn near the fovea (Figure 1B). Horizontally oriented optical coherence tomography revealed an increased macular thickness and an accumulation of intraretinal fluid, indicating macular oedema (Figure 1C). After three intravitreal injections of 1.25 mg bevacizumab, her vision improved to 20/20. Ophthalmoscopic examination revealed a decreased calibre of the previously engorged draining veins and ghost vessels (Figure 1D). Repeated fluorescence angiography failed to show fluorescence leakage (Figure 1E), and horizontally oriented optical coherence tomography revealed a decreased macular thickness and the absence of an intraretinal cyst (Figure 1F). At the 2-year follow-up visit, the vision of the patient was stable.

## Conclusion

In the present case, we chose to use anti-VEGF because we observed fluorescence leakage and retinal oedema during optical coherence tomography. Although the vision of the patient improved to 20/20 and remained stable for 2 years after the three intravitreal injections of bevacizumab, the regression of the abnormal, engorged mature vessels after treatment was unexpected. This thromboembolic event can be explained by either spontaneous involution or induced platelet aggregation followed by degranulation and thrombosis through the formation of a complex with VEGF and activation of the platelet FcγRIIa receptor [6]. This finding represents the case-report of a single individual and must be studied more extensively before it can be generalised on a larger basis or used to determine a standard of care. However, clinicians should be cautious of the potential thrombotic effects on systemic blood vessels when administering anti-VEGF treatment.

## Consent

Written informed consent was obtained from the patient for the publication of this case report and any accompanying images. A copy of the written consent is available for review by the Editor of this journal.

## Abbreviations

VEGF: Vascular endothelium growth factor.

## Competing interests

The authors declare that they have no competing interests. Chi-Chun Lai is a current consultant of the pharmaceutical corporations Allergan, Bayer, and Novartis. The other authors have no financial disclosures.

## Authors’ contributions

Conceiving and designing the study (CCL, LHC); data collection (NKW, YPC); analysing and interpreting the data (NKW, WCW); writing the manuscript (LHC), providing critical revisions (YPC, WCW) and approving the final version (CCL, LHC, NKW, YPC, WCW). All authors read and approved the final manuscript.

## Pre-publication history

The pre-publication history for this paper can be accessed here:

http://www.biomedcentral.com/1471-2415/13/60/prepub
